# Shared influence of pathogen and host genetics on a trade-off between latent period and spore production capacity in the wheat pathogen, *Puccinia triticina*

**DOI:** 10.1111/eva.12000

**Published:** 2012-09-07

**Authors:** Bénédicte Pariaud, Femke Berg, Frank Bosch, Stephen J Powers, Oliver Kaltz, Christian Lannou

**Affiliations:** 1 UMR1290 Bioger, INRA, BP 01F-78850, Thiverval Grignon, France; 2 Department of Computational and Systems Biology, Rothamsted ResearchHarpenden, Hertfordshire AL5 2JQ, UK; 3 Institut des Sciences de l'Evolution, UMR CNRS-UM2-IRD5554, CC 065, Université Montpellier 2Place E. Bataillon, 34095 Montpellier, Cedex 05, France

**Keywords:** genetic correlation, latent period, leaf rust, sporulation capacity, *Triticum aestivum*, Wheat

## Abstract

Crop pathogens are notorious for their rapid adaptation to their host. We still know little about the evolution of their life cycles and whether there might be trade-offs between fitness components, limiting the evolutionary potential of these pathogens. In this study, we explored a trade-off between spore production capacity and latent period in *Puccinia triticina*, a fungal pathogen causing leaf rust on wheat. Using a simple multivariate (manova) technique, we showed that the covariance between the two traits is under shared control of host and pathogen, with contributions from host genotype (57%), pathogen genotype (18.4%) and genotype × genotype interactions (12.5%). We also found variation in sign and strength of genetic correlations for the pathogen, when measured on different host varieties. Our results suggest that these important pathogen life-history traits do not freely respond to directional selection and that precise evolutionary trajectories are contingent on the genetic identity of the interacting host and pathogen.

## Introduction

Basic theory of parasite evolution relies on the existence of negative relationships (trade-offs) between characters determining parasite fitness. For example, trade-offs between parasite virulence and transmission capacity may explain why parasites evolve to cause intermediate levels of damage to their hosts (Alizon et al. 2009). Trade-offs between parasite life-history traits may also facilitate the maintenance of different genetic variants in parasite populations (Héraudet et al. 2008) and thereby influence the severity of epidemics. Understanding the nature and strength of trade-offs therefore provides important insights in the constraints of parasite evolution, a question central to disease management (Dieckmann et al. 2002).

Parasite life cycles consist of a sequence of elementary quantitative traits, such as infection efficiency, within-host growth, latent period, spore production and transmission (Ennos 1991; Pariaud et al. 2009a; Lannou 2012). These traits are likely to be under constant direct selection imposed by the host's defence system. Genetic or evolutionary trade-offs arise if an increase in one trait is linked with a decrease in the other, and therefore, fitness cannot be maximized for both traits. Thus, negative correlations between these components represent a potential evolutionary constraint for the parasite. Commonly studied trade-offs involve relationships between within-host development and between-host transmission, often with consequences for host fitness (virulence). Traits facilitating establishment of infection can impair subsequent within-host growth (Sacristan and Garcia-Arenal 2008; Bahri et al. 2009), and in turn, within-host growth rate can impact timing and quantity of reproductive output (spore production). This can generate virulence-transmission trade-offs (Lipsitch and Moxon 1997; Ebert and Bull 2003; Alizon et al. 2009) or latent period-spore production trade-offs (Héraudet et al. 2008; Nidelet et al. 2009).

Although described for various systems, trade-offs are far from being ubiquitous, and their importance for parasite evolution remains debated (Ebert and Bull 2003; Sacristan and Garcia-Arenal 2008; Alizon et al. 2009). In particular, recent studies indicate context-dependent variation of traits involved in host–parasite interactions (Laine 2008; Wolinska and King 2009; Hall et al. 2010), and this may also affect the phenotypic and genetic correlation between these traits. Thus, trade-off shapes may vary with environmental conditions (Vale et al. 2011) or with genetic background. Namely, if trait expression is under the shared control of host and parasite (Restif and Koella 2003; Lambrechts et al. 2006), correlations may depend on the interaction between host and parasite genotypes (Salvaudon et al. 2005; de Roode and Altizer 2010).

Heterogeneity in these trade-off relationships is commonly analysed by means of analysis of covariance (ancova) (e.g. Salvaudon et al. 2005; Magalon et al. 2010; de Roode and Altizer 2010). By regressing one trait on the other, it can be tested whether the slope of the relationship varies with factors of interest, such as parasite or host genotype, environmental factors or selection treatments. However, as pointed out by Graham et al. (2011), one limitation of this approach is the assumption of a causal relationship between the two traits. Alternatively, one may use both traits as response variables in a multivariate analysis of variance (manova), an approach that has only recently been applied in a parasite trade-off context (Vale et al. 2011; see also Mideo et al. 2011). The manova models the covariance between the two traits rather than the variance in one trait as a function of the other. One can therefore estimate how much of the covariance, that is, of the trade-off, is explained by the factors of interest (parasite genotype, host genotype, etc.). This technique is particularly convenient for hierarchically structured data, for example, when trade-offs are to be compared within and between geographic or genetic origins (He et al. 2009).

Here we illustrate how a combined approach of ancova and manova can be used to investigate a life-history trade-off in *Puccinia triticina* Eriks., a fungal pathogen causing leaf rust of wheat (*Triticum aestivum* L.). It is the most common rust disease of wheat, causing substantial yield loss in all production areas of the world (Bolton et al. 2008). In crop systems, pathogen evolution is driven by human intervention, constantly introducing and removing different plant varieties. Varieties are well characterized for their resistance factors, and techniques are available to accurately measure quantitative traits in the pathogen. This provides interesting opportunities to study pathogen life-history evolution and adaptation to their hosts. Knowledge of the genetic architecture of life-history traits in crop pathogens may also guide resistance management, as it can help predict the epidemiological spread of particular pathotypes (Thrall et al. 2011). Nonetheless, still surprisingly little is known about the variation and covariation in these traits in fungal pathogens, and how relationships are modulated when traits are expressed on different host varieties.

Pariaud et al. (2009b) investigated quantitative variation in pathogenicity-related traits for French leaf rust pathotypes. From this database, we identified a phenotypic trade-off between two pathogen life cycle components: latent period (time interval between infection and onset of spore production) and spore production capacity (SPC). Analogous to ‘age at maturity’ in nonparasitic organisms, the latent period relates to the rate at which the parasite develops in the host tissues (Nelson 1979), thereby determining the time-lag between subsequent pathogen generations. As *P. triticina* causes polycyclic epidemics, the latent period is a crucial fitness determinant of this pathogen (Lehman and Shaner 1997; Lannou 2012). SPC refers to the amount of spores produced per unit area of sporulating tissue per time unit (Pariaud et al. 2009b). It reflects the pathogen's competence of exploiting host resources and converting them into spores.

We analysed previously published data from two experiments by Pariaud (2008), Pariaud et al. (2009b), measuring latent period and SPC for fungal isolates from different pathotypes on different host genotypes (varieties). Using the manova approach described by He et al. (2009), we analysed how much of the covariance between the two traits was explained by the genetic factors of pathotype, isolate within pathotype and host varietyand the interaction between these factors. Using the common ancova approach, we tested whether the strength (i.e. the slope) of the trade-off varied with the identity of pathotype or host variety.

## Materials and methods

### Study organism

The basidiomycete *P. triticina* (Uredinales) is highly specialized to common wheat and durum wheat and has a worldwide distribution (Bolton et al. 2008). Leaf rust survives between crops as mycelium or as uredinia on infected volunteer and/or on early-sown and late-maturing wheat crops. Urediniospores are wind-dispersed over large distances. The short uredinial infection cycle (7–10 days) allows the building up of multiple asexual (clonal) generations and thus the rapid spread of infection within a single wheat-growing season. All populations of *P. triticina* thus far examined show very strong indications of clonal reproduction and no evidence for sexual recombination (Kolmer et al. 2011). Repeated introduction of host resistance genes, operating according to a gene-for-gene relationship with pathogen avirulence genes (Flor 1971), exerts strong selection on the pathogen and has led to a ‘pathotype’ population structure. Different pathotypes carry different combinations of avirulence genes and are characterized by their capacity to infect or not wheat differential lines with specific resistance genes. Isolates of the same pathotype are highly homogeneous for microsatellite markers (Goyeau et al. 2007), suggesting close common ancestry. Pathotypes are defined on the basis of qualitative characters (ability to infect or not). Nonetheless, differences can be found for quantitative traits within a pathotype (Pariaud et al. 2012), indicating relevant genetic variation below the pathotype level.

### Experimental design

Data from two inoculation experiments (Pariaud 2008) were used for the present analysis. Experiment 1 employed two isolates from each of the three pathotypes (P1, P2 and P3) collected from different locations in France between 1997 and 2002 (French wheat leaf rust national survey, Goyeau et al. 2006). Pathotype P1 and P2 isolates were collected from the wheat variety *Soissons*, and P3 isolates from the variety *Trémie* (for their virulence gene profiles, see Table 1).The main objective of this experiment (Pariaud et al. 2009b) was to test for within-pathotype variability, and therefore, the performance of each isolate was measured with high precision (12–15 replicates) on a single host variety (Soissons). The two isolates sharing the same pathotype had identical microsatellite profiles but had shown potentially contrasting sporulation capacities in preliminary tests. Experiment 2 focused on the combined effects of host variety and pathotype identity on the expression of pathogen life-history traits. Two pathotypes (P1 and P2) were tested on five wheat varieties (namely Soissons, Festival, Morocco, Scipion and Thesee). The choice of the pathotypes in experiment 2 was not based on the results of experiment 1 as they were not available yet at this time. Six isolates per pathotype were used, with mostly five replicates per isolate/variety combination (Table 1). Note that the experimental design was not fully factorial because certain varieties are fully resistant to certain pathotypes.

**Table 1:** Cross-inoculation design of experiment 2

	Host variety (resistance gene)				
	
Pathotype-isolate	Morocco (–)	Soissons (Lr14a)	Thésée (Lr13)	Festival (–)	Scipion (–)
P1-a	5	5	–	5	5
P1-b	5	4	–	–	5
P1-c	5	5	–	3	4
P1-d	5	3	–	5	4
P1-e	4	3	–	2	4
P1-f	4	5	–	5	4
P2-g	5	5	5	–	–
P2-h	5	5	5	–	–
P2-i	5	5	5	–	–
P2-j	5	5	5	–	–
P2-k	4	5	5	–	–
P2-l	5	5	5	–	–

Six isolates (a–l) from each of two pathotypes (P1, P2) were tested on five host varieties. For each of the 41 isolate – host variety combinations, the number of replicates is shown. Three pathotype – variety combinations could not be tested because the varieties were fully resistant to these pathotypes: P1 is not able to overcome the Lr13 resistance gene of Thésée and Festival and Scipion expressed an adult-type resistance to P2, with a very limited and delayed expression of the symptoms.

### Experimental protocols

Experiments were performed in a greenhouse on adult wheat plants, grown under standardized conditions. In each experiment, one replicate consisted of one pot containing one main wheat stem, of which the flag leaf was inoculated. Isolate maintenance and inoculation procedure are described in detail elsewhere (Pariaud et al. 2009b). All inoculations were performed with freshly produced uredospores. The plants were inoculated at the heading or flowering stage by brushing spores with a soft brush on leaf sections of 8–9 cm in length. Latent period and SPC were measured on the same leaf.

Sporulation starts when uredia rupture the leaf epidermis. However, not all lesions on the same leaf start sporulating simultaneously (Shaner 1980). Therefore, for 8 days, a 1-cm² inoculated leaf section was checked twice a day for new sporulating lesions. The latent period was taken as the time (in degree-days) until 20% of the maximum number of lesions was sporulating (LP20). This time was estimated by linear interpolation around the lesion counts flanking 20% (Knott and Mundt 1991). Other authors sometimes use a 50% threshold, but we chose LP20 to better capture the time of first spore production on a leaf.

During the sporulation period, the leaves were placed in open plastic tube to collect the spores (see Pariaud et al. 2009b). We were thus able to collect and weigh all the spores produced between days 15 and 21 after inoculation. Image analysis was carried out on the digital photographs of the leaves to determine the sporulating surface area during the same period (Optimas 5; Media Cybernetics, Silver Spring, MD, USA). SPC was calculated as the total weight of spores produced divided by the average sporulating surface area and by the number of days (μg of spores mm^−2^ day^−1^). We chose days 15–21 as sampling period because it allows reliable measurements of both spore production and lesion size (Robert et al. 2004).

### Statistical analysis

Spore production capacity decreased with lesion density (experiment 1: *r* = −0.51, *n* = 120, *P* < 0.0001; experiment 2: *r* = −0.46, *n* = 188, *P* < 0.0001). To account for this density dependence, we carried out a linear regression of SPC on lesion density and used the regression model to estimate SPC values at a fixed density (the median density of 28 lesions cm^−2^), as in (Lannou and Soubeyrand 2012). These standardized SPC values were used for further analysis and figures. There was no significant density dependence for latent period.

Following He et al. (2009, p. 2781), we performed multivariate analysis of variance *sensu* Kempthorne (1969) to investigate the influence of host and pathogen identity on the trade-off between latent period and SPC. This type of MANOVA uses ordinary least square techniques, rather than REML procedures, to calculate sums of products (SP) describing the covariance explained by the different factors in the statistical model. SP are analogous to sums of squares (SS) in an anova and can be calculated from eqn (1):


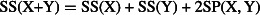
(1)

where X and Y are the two traits, and X + Y is the sum of the two traits. Mean sum of products (SP/degree of freedom) were used to calculate *F* values for hypothesis testing. Furthermore, because SP are additive, we were able to calculate covariance components (% of SP explained by a given factor in the model), analogous to variance components in an anova. Model fitting was done in a SAS-type II fashion (SAS 1996), that is, the SP of a given factor in the model was obtained after fitting all other terms not containing this factor.

For the data from experiment 1, the manova model included the effects of pathotype and isolate nested within pathotype. For the data from experiment 2, we first considered the 41 combinations of pathogen isolate and host variety as independent groups in the analysis. We then decomposed the covariance into components explained by host or pathogen identity (Table 1). A significant ‘pathotype’ effect in the manova would mean that some pathoypes have a long latency period and high SPC, while others have a short latent period and low SPC.

For analyses of covariance (ancova), we calculated means of SPC and LP20 per isolate (*n* = 6 means in experiment 1) and per isolate × host variety combination (*n* = 41 means in experiment 2, see Table 1). We chose mean SPC as the response variable and mean LP20 as the covariate. Inverting the roles of SPC and LP20 gave very similar results (not shown). Cofactors were represented by pathotype identity, isolate nested within pathotype, host variety or isolate by variety combination. We also fitted cofactor × LP20 interactions to test for variation in slope among the different genetic factors. A significant interaction would indicate that the strength of the trade-off varies with host or pathogen identity. In initial models, second-order terms of LP20 were always nonsignificant (*P* > 0.2), and there was thus no evidence for nonlinearity in the relationship between latent period and SPC. For all analyses, latent period was square-root-transformed, standardized SPC log-transformed. We used the SAS (1996) and JMP (SAS 2009) statistical packages for analysis.

## Results

### Experiment 1

**Figure 1:**
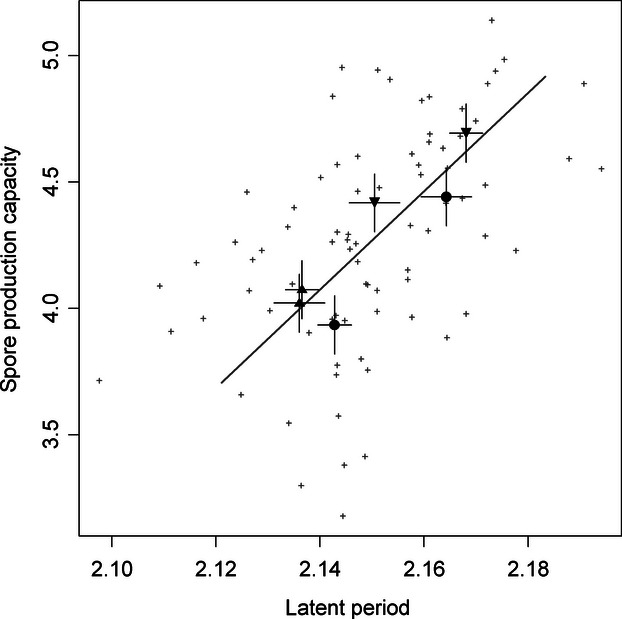
Data from experiment 1. Latent period (LP20, log-transformed) and spore production capacity (SPC, square-root transformed) of two isolates of each of the pathotypes P1 (circles), P2 (triangles) and P3 (reversed triangles). The regression line is made on the isolate means and shows the genetic trade-off. Standard error for the mean values are indicated. Each isolate was replicated 15 times (crosses).

Overall, there was a significant positive correlation (Pearson correlation coefficient, *r* = 0.50, *n* = 82, *P* < 0.0001) between latent period and SPC. As short latent period and high sporulation capacity represent fitness advantages to the fungus, this positive correlation establishes a phenotypic trade-off between the two traits (Fig. 1).

The manova (Table 2) showed that pathogen identity accounted for 80% of the covariance (i.e. of the total sum of products) between the two traits. Thus, the trade-off had a strong genetic component. Both pathotype and isolate identity contributed to the covariance, but because of the small number of degrees of freedom in the *F*-test the pathotype effect was only marginally significant.

**Table 2:** manova for experiment 1

Source	df	SP	% SP*	*F*	*P*
Pathotype	2	0.1547	48.2	2.25	0.0893
Isolate [pathotype]	3	0.1030	32.1	41.19	<0.0001
Residual	76	0.0634	19.7		

The pathotype effect was tested over the isolate [pathotype] effect.

* %SP: proportion of the total Sum of Products (covariance) explained by each factor.

Across the six isolate means, we obtained a highly positive correlation between the two traits (*r* = 0.91, *n* = 6, *P* = 0.0127), confirming the strong genetic trade-off: isolates with a short latent period had a reduced SPC. The effect of latent period was still significant (*F*_1,2_ = 47.32, *P* = 0.0205), when accounting for pathotype identity in an ancova. This means that the trade-off also held across isolates within pathotypes, consistent with the significant isolate effect in the manova (Table 2).

### Experiment 2

**Figure 2:**
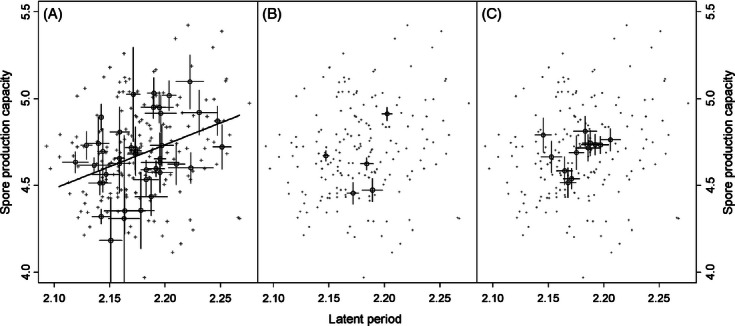
Data from experiment 2. Latent period (LP20, log-transformed) and spore production capacity (SPC, square-root transformed) of 41 combinations of five host varieties and 12 pathogen isolates (six of pathotype P1 and six of pathotype P2). (A) Mean values for each isolate – variety combination (circles) with standard errors. The regression line is made on the means and shows the genetic trade-off. (B) Mean values for each variety (circles). (C) Mean values for each isolate (circles). Each isolate – variety combination was replicated five times (crosses).

As in experiment 1, there was a significant positive phenotypic correlation between latent period and spore production (*r* = 0.17, *n* = 188, *P* = 0.0184; Fig. 2A).

In the manova (Table 3), 87% of the covariance between the two traits was explained by the combination of pathogen and host genetic identity (41 variety-isolate combinations; Fig. 2A). Further decomposition of this covariance revealed a very strong host variety contribution. Thus, a large part of the correlation was owing to some varieties causing a long latent period and high spore production, and others causing a short period and low spore production (Fig. 2B). The manova further revealed significant pathotype and isolate main effects (Fig. 2C), but the two factors together only explained <20% of the covariance. An even smaller, albeit still significant, portion of the covariance (<13%) was explained by the combined influence of pathogen and host genetic factors (pathogen × host variety interactions).

**Table 3:** manova for experiment 2

Source of variation	df	SP	% SP*	Denom.†	*F*	*P*
Overall						
Combination isolate-variety	40	0.4393	86.9	(1)	24.37	<0.0001
Residual (1)	147	−0.0663	13.1			
Decomposition						
Host variety	4	0.3148	57.0	(3)	43.11	<0.0001
Pathotype	1	0.0426	7.7	(2)	7.18	0.0231
Isolate [pathotype] (2)	10	0.0593	10.7	(3)	3.25	0.0087
Pathotype × variety	1	−0.0256	4.6	(3)	14.04	0.0001
Isolate × variety [pathotype] (3)	24	0.0438	7.9	(1)	4.05	<0.0001

SP, Sums of products. The SP explained by the 41 combinations of pathogen isolate and host variety was decomposed into individual contributions of pathotype, isolate and host variety, and their interactions.

* The %SP column shows the proportion of the total (summed over absolute values of SP) Sum of Products (covariance) explained by each factor.

† The Denominator (Denom.) column denotes the error terms used for the *F*-tests.

**Figure 3:**
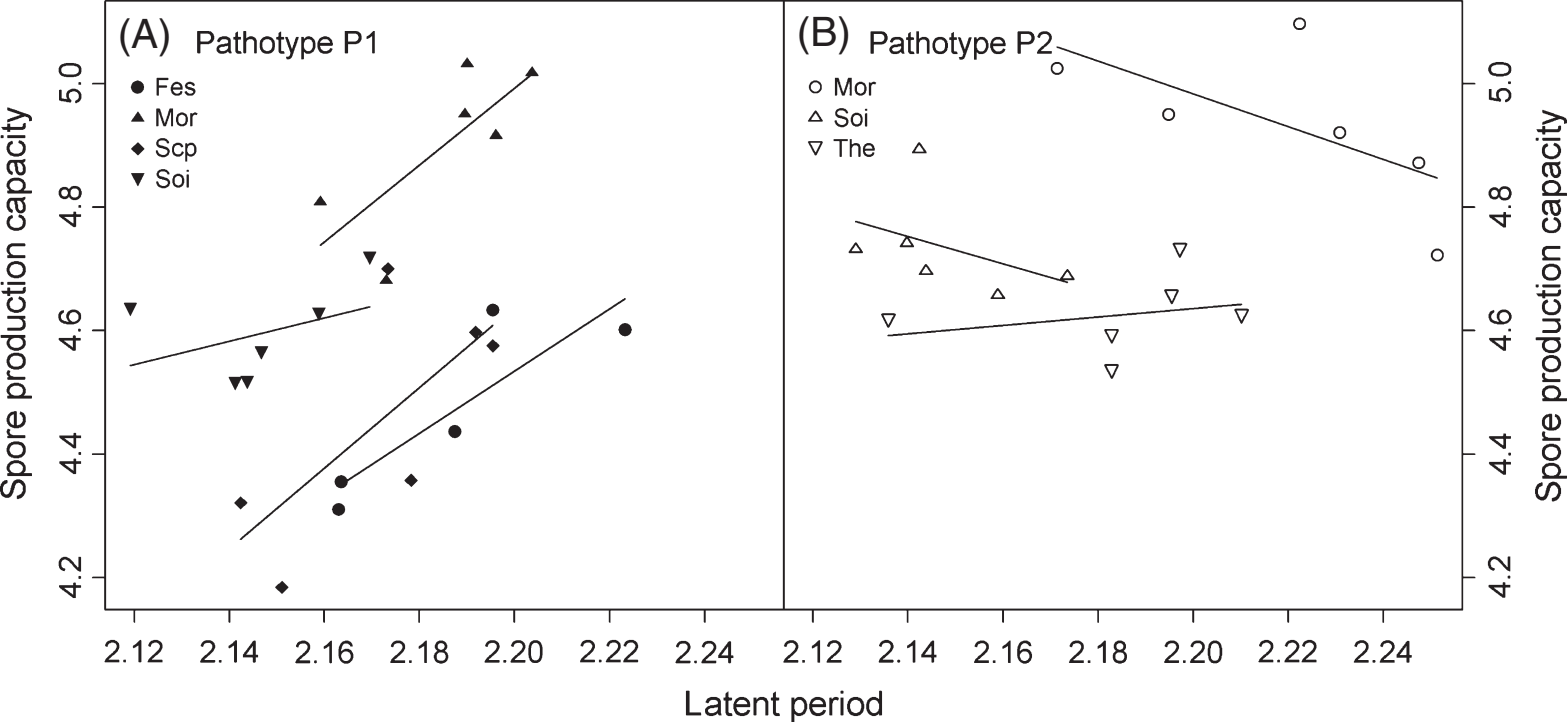
Data from experiment 2. Latent period (LP20, log-transformed) and spore production capacity (SPC, square-root transformed) of 41 combinations of five host varieties and 12 pathogen isolates (six of pathotype P1 and six of pathotype P2). Each point is the average value of five replications. The continuous lines show regressions for each variety – pathotype combination. The different varieties are indicated in the figure. (A) Pathotype P1; (B) pathotype P2.

For the means of the 41 combinations of isolate and host variety, we obtained a significant positive correlation between latent period and SPC (*r* = 0.37, *n* = 41, *P* = 0.0163), confirming the genetic contribution to the trade-off (Fig. 3). Because the experimental design was not fully factorial, we first carried out a simplified ancova, with the seven pathotype-host variety combinations (Table 1) as independent groups of points. For these groups, we found significant differences in the slope of the latent period-spore production relationship (latent period × combination interaction, Table 4). For more detailed analysis, we decomposed the variance into contributions from host variety and pathotype. This revealed a significant latent period × pathotype interaction (Table 4): Pathotype P1 showed relatively strong positive correlations between latent period and spore production and thus genetic trade-offs, on all four host varieties (Fig. 3A). In contrast, pathotype P2 produced weakly positive or even negative correlations (Fig. 3B).

**Table 4:** ancova for experiment 2

Source of variation	df	MS	*F*	*P*
Overall				
Latent period	1	0.0671	6.42	0.0174
Pathotype-variety combination	6	0.1414	13.54	<0.0001
Latent period × combination	6	0.0371	3.56	0.0100
Error	27	0.0104		
Decomposition				
Host variety	4	0.2096	8.42	0.1090
Pathotype [variety]	2	0.0249	2.38	0.1114
Latent period × variety	4	0.0214	2.05	0.1156
Latent period × pathotype [variety]	2	0.0468	4.49	0.0208

The overall variance explained by the seven pathotype-host variety combinations was decomposed into the individual contributions of pathotype and host variety. Because the experimental design was not fully factorial, pathotype was nested within variety. Thus statistical tests compared the two pathotypes only on the varieties on which they were both assayed. Host variety was tested over the pathotype [variety] effect, all other terms over the Error term.

## Discussion

Basic concepts in evolutionary biology assume that trade-offs between fitness components constrain the response to selection and thereby prevent the fixation of omnipotent genotypes. Most of the published data on trade-offs, however, concern microparasites (see Alizon et al. 2009), and still, little is known about how such trade-offs might constrain the evolution of agronomically relevant pathogens (mainly fungi and oomycetes) or guide plant breeding and resistance management. Our study on the wheat rust pathogen, *P. triticina*, revealed a classic life-history trade-off, namely between age at maturity (latent period) and fecundity (SPC). This indicates that faster colonization of host tissue and building up of sporulation structures is achieved at the expense of the quantity of spores produced in these structures. Such a cost of early reproduction is also known in other parasitic organisms, such as bacteriophages, where early lysis time is associated with smaller burst size (Wang 2006).

### Host and pathogen genotype exert shared control of the trade-off

We found a genetic basis of the trade-off with contributions from both host and pathogen. In experiment 1, using a single host variety, pathogen genetic identity accounted for 80% of the covariance between latent period and spore production. This means that isolates and pathotypes can be ranked along the trade-off, with some reproducing more quickly, but with relatively fewer spores, and others developing more slowly, but producing more spores. Experiment 2, employing different combinations of pathogen and host genotypes, further revealed a substantial contribution from host genotype and, to a lesser extent, interactions between host and pathogen genotype. The contribution of the pathogen to the trade-off was lower in experiment 2 maybe because only P1 and P2 were used in that experiment. Both Figs 1 and 3 suggest that the isolates of P2 contributed less to the trade-off than those of P1 and P3.

Using a natural plant–pathogen system (*Hyaloperonospora parasitica* – *Arabidobsis thaliana*), Héraudet et al. (2008) also found a phenotypic trade-off between latent period and transmission success. There was no evidence for a genetic trade-off, possibly because only a small set of genotype combinations was tested. Nevertheless, their experiment showed that transmission of certain combinations of host plant and pathogen genotypes responded differently to variation in the latent period (Héraudet et al. 2008).

Interestingly, we obtained significant genetic correlations across isolates with the same pathotype. Because pathotypes typically show strong clonal structure (Goyeau et al. 2006) phytopathologists often consider the pathotype as basic unit to describe *P. triticina* populations. Our findings, however, indicate that selection for quantitative traits can operate at the isolate level, independently of the well-documented selection for qualitative virulence factors at the pathotype level (see also Pariaud et al. 2012). It is also consistent with the idea that trade-offs can play a role in evolutionary processes at a relatively small, possibly local, scale in this pathogen.

Shared control of the covariance between latent period and SPC was not only attributable to pathogen and host genotype main effects; the manova also revealed significant pathogen genotype × host genotype interactions. That is, the rank order of pathogen isolates or pathotypes along the trade-off changed with host variety. Similarly, there also was a significant higher-order interaction between covariate, pathotype and host variety in the ancova (Table 4). Thus, the (across-isolate) genetic correlations differed between the two pathotypes: the trade-off was strong for pathotype P1 isolates, but absent for P2 isolates, with variable sign and strength of these relationships according to the host varieties tested (Fig. 3).

Salvaudon et al. (2007) reported similar results for the relationship between pathogen fitness (transmission) and host fitness (seed production) in the above-mentioned pathogen of *Arabidobsis*. Genetic correlations in the pathogen were differently expressed on different host genotypes, although, unlike in our study, there was no significant overall relationship between the two traits for all combinations confounded. The rank order of parasite genotypes along a virulence-transmission trade-off also changed when tested on different host genotypes of Monarch butterflies (de Roode and Altizer 2010). These observations highlight the potentially complex genetic determinism of traits involved in host-parasite interactions.

### Genetic determinism of the trade-off

As advocated by Lambrechts et al. (2006), the phenotype of a host-pathogen interaction is likely to be controlled by the genotype of both players. Our results, along with others (Salvaudon et al. 2007), extend this view in that not only individual traits, but also genetic correlations among these traits are under the shared control of host and parasite. Recent work on disease resistance in cultivated plants has begun to unveil the underlying genetic basis. Indeed, quantitative trait loci (QTL) analyses have shown that the phenotype of plant – pathogen interactions can be determined by the host genotype (e.g., Ballini et al. 2008) as well as by the pathogen genotype (Cumagun et al. 2004; Lind et al. 2007). QTL analyses also corroborate the idea that genetic correlations are influenced by the host genotype × pathogen genotype interaction since QTLs can be isolate specific, that is, can respond to certain isolates, but not to others (Calenge et al. 2004; Marcel et al. 2008).

In studies on plant quantitative resistance to diseases, pathogenicity traits are sometimes found to be positively correlated and under the pleiotropic control of resistance genes (Milus and Line 1980; Lehman and Shaner 1997, 2007). This is, however, not in contradiction with the existence of trade-offs between quantitative traits (Lannou 2012). As pointed out by Pease and Bull (1988), a trade-off has to be considered with regards to a constant resource. Thus, different host genotypes can be seen as different environments for the pathogen, with variable resource availability (see also Salvaudon et al. 2007). The presence of a QTL for resistance with pleiotropic effects represents a resource limitation for the pathogen and this might explain why in some cases, positive correlations are found among quantitative traits instead of trade-offs. For example, in a study on another rust fungus of wheat (*Puccinia striiformis* f.sp. *tritici)*, fungal isolates with a shorter latent period also produced more spores. A positive relationship, rather than a trade-off, was also observed after experimental evolution of a bacterial parasite of *Paramecium* (Nidelet et al. 2009). These contrasting findings demonstrate that trade-offs between life-history traits, although conceptually widely accepted, are far from being ubiquitous.

### Implications for pathogen evolution and host resistance management

Multiple infection cycles boost wheat rust epidemics, and therefore, strains with the shortest latent period should have a selective advantage and predominate seasonal disease progression (Lehman and Shaner 1996; Sackett and Mundt 2005). However, our results indicate that directional selection for shorter latent period can be offset by a trade-off with SPC. Generally, trade-offs facilitate the maintenance of genetic variation in the traits involved (Roff and Fairbairn 2007). Whether the observed trade-off facilitates coexistence of pathogen genotypes with contrasting latent periods and spore production capacities depends on how the two traits contribute precisely to total fitness. For example, if fitness is maximal for intermediate values of the traits (see Wang 2006), one would predict fixation of a single optimal pathogen genotype rather than coexistence of multiple genotypes.

Predicting evolutionary outcomes may be even more difficult, given the shared genetic control of the trade-off by pathogen and host. Namely, the variable strength (and even sign) of the genetic correlations on different host varieties suggests that the trade-off constrains selection on some hosts, but not on others. Furthermore, rank order changes along the trade-off may favour different pathogen genotypes on different host varieties. As a result, geographic mosaics (Thompson 2006) may arise, with different evolutionary trajectories for these life-history traits occurring in individual wheat fields or production areas.

Finally, our results have potential implications for the choice of host varieties by plant breeders or farmers. The existence of trade-offs between quantitative traits and the variability of patterns for different host – pathogen combinations is consistent with the idea that diversifying the genetic background and the QTLs for quantitative resistance of the varieties grown in agricultural landscapes would hamper the evolution of the pathogens towards higher virulence (Papaïx et al. 2011) and contribute to the durability of resistance (Lehman and Shaner 1997; Lannou 2012).

## Conclusions

We demonstrated a genetic basis of a trade-off between two life-history traits (latent period and spore production), potentially limiting the evolution of this pathogen. A simple multivariate (manova) technique allowed us to quantify the relative contributions of host and pathogen genotype to the trade-off. We showed that the covariance between traits, just like the variance, can be under shared control of host and pathogen, with contributions via host and pathogen genotype main effects and interactions.

There was a clear overall correlation for all host–pathogen genotype combinations pooled, but we also found that sign and strength of genetic correlations for the pathogen varied on different host varieties. This pattern illustrates how opposing trends can be embedded in an overall relationship between two traits, a common theme in life-history theory (Stearns 1992). It also reflects the general observation that natural selection may ‘see’ variable amounts of genetic variation and covariation in different environments (Blanckenhorn and Heyland 2004), here the ‘environment’ being host genotype.

Finally, our results have potential implications for the choice of host varieties by plant breeders or farmers. It is often expected that quantitative resistance pleiotropically affect the different components of the pathogen cycle. Thus more resistant varieties, prolonging the onset of disease symptoms, should also be better at inhibiting spore production. Here, however, we observed the opposite pattern: latent period and SPC were positively correlated across the five host varieties. This suggests a more complex genetic architecture of quantitative resistance genes, which is sustained by recent QTL analyses. We believe that taking into account this complexity can help better understand the factors driving pathogen evolution. Including trade-offs in disease management models may greatly improve our capacity to control disease in the long term in agrosystems (van den Berg et al. 2010).

## Data archiving statement

Data for this study are available as Supporting Information attached to the online version of the publication.

## Supporting Information

**Data S1.** Pathotype and isolate identification; latent period (T20, in degree-days) and spore production capacity (spm, in micro-grams/mm^2^).

**Data S2.** Pathotype, isolate and variety identification; latent period (T20, in degree-days) and spore production capacity (spm, in micro-grams/mm^2^).

**Table S1**. Identity of resistance genes overcome by the three pathotypes (P1, P2, P3) used in our experiments, as determined on differential host lines.
