# Supplementary material for: Shared influence of pathogen and host genetics on a trade-off between latent period and spore production capacity in the wheat pathogen, Puccinia triticina
Source: Evol Appl. 2012 Sep 7;6(2):303–12. doi: 10.1111/eva.12000 (PMC3586619; doi:10.1111/eva.12000)
Supplement: Supplementary file 3 [file eva0006-0303-SD3.docx]

**Supplementary Materials.**

**Table S1**. Identity of resistance genes overcome by the three pathotypes (P1, P2, P3) used in our experiments, as determined on differential host lines.

|  | Resistance genes |
| --- | --- |
| P1 | *Lr2c, Lr3, Lr3bg, Lr3ka, Lr10, Lr14a* |
| P2 | *Lr2c, Lr13, Lr14a, Lr26, Lr17b* |
| P3 | *Lr1, Lr3, Lr3bg, Lr10, Lr13, Lr14a, Lr15, Lr17, Lr17b, Lr20, Lr37, Lr27+Lr31* |
